# Combined High Hydrostatic Pressure and Additive Chemical Treatment Enhances Decontamination Efficiency in Bone Tissue Infected with Staphylococcal Biofilms

**DOI:** 10.3390/microorganisms14071502

**Published:** 2026-07-09

**Authors:** Henrike Loeffler, Adam Bieda, Martina Sombetzki, Ralf-Joerg Fischer, Mirko Basen, Karoline Schulz, Janine Waletzko, Rainer Bader, Annett Klinder

**Affiliations:** 1 Biomechanics and Implant Technology Research Laboratory, Department of Orthopedics, Orthopedic Clinic and Polyclinic, Rostock University Medical Center, 18057 Rostock, Germany; janine.waletzko-hellwig@med.uni-rostock.de (J.W.); rainer.bader@med.uni-rostock.de (R.B.); annett.klinder@med.uni-rostock.de (A.K.); 2 Department for Internal Medicine, Clinic and Polyclinic for Infectious Diseases, Nephrology, Endocrinology and Tropical Medicine, Rostock University Medical Center, 18057 Rostock, Germany; adam.bieda@med.uni-rostock.de (A.B.); martina.sombetzki@med.uni-rostock.de (M.S.); 3 Microbiology, Institute of Biological Sciences, University of Rostock, 18059 Rostock, Germany; ralf-joerg.fischer@uni-rostock.de (R.-J.F.); mirko.basen@uni-rostock.de (M.B.); 4 Department Maritime Systems, Faculty of Interdisciplinary Research, University of Rostock, 18059 Rostock, Germany; 5 Medical Biology and Electron Microscopy Centre, Rostock University Medical Center, 18057 Rostock, Germany; karoline.schulz2@med.uni-rostock.de

**Keywords:** high hydrostatic pressure, *Staphylococcus aureus*, *Staphylococcus epidermidis*, bone graft, sterilization, osteomyelitis

## Abstract

Osteomyelitis remains one of the most persistent challenges in orthopedic and trauma surgery, often requiring removal of infected bone tissue segments and leaving critical size defects. Reimplantation of sterilized autologous bone may combine optimal anatomical fit with biological compatibility. This study investigates sodium dodecyl sulfate (SDS) and ethylenediaminetetraacetic acid (EDTA) in combination with high hydrostatic pressure (HHP) as a physicochemical sterilization approach for *Staphylococcus aureus* and *Staphylococcus epidermidis*, two of the predominant pathogens associated with osteomyelitis. SDS/EDTA dose determination assays were performed prior to inactivation of different staphylococcal species via HHP with additives. Planktonic *S. aureus* ATCC 35556 and *S. epidermidis* ATCC 35984 and ATCC 12228 were exposed to HHP (250 MPa and 350 MPa) in pressure-transmitting media with SDS and EDTA. Furthermore, inactivation of *S. aureus* and *S. epidermidis* within biofilms on human trabecular bone cylinders was quantified based on logarithmic reduction factors and evaluated using scanning electron microscopy (SEM). Under HHP, planktonic *S. aureus* displayed pronounced pressure tolerance, whereas both *S. epidermidis* strains were fully inactivated at 350 MPa even in 0.9% saline. The addition of SDS/EDTA, which exhibited additive effects (fractional inhibitory concentration (FIC) = 0.7) in planktonic *S. aureus*, significantly enhanced pressure-induced killing, particularly in biofilms typically limiting sterilization efficiency. SEM revealed substantial membrane deformation and cell wall disruption following the treatment. HHP combined with SDS/EDTA effectively inactivated planktonic and biofilm-associated staphylococci, providing a promising strategy for safe autologous bone reimplantation after infection with the potential to improve clinical outcomes.

## 1. Introduction

Osteomyelitis, an infection of bone and bone marrow, represents a major challenge in orthopedic and trauma surgery and can lead to serious complications if not effectively treated [1,2,3,4]. Recent data indicate a rising incidence of osteomyelitis in Germany with a 10.4% increase in prevalence between 2008 and 2018, reaching 16.7 cases per 100,000 inhabitants [5].

Clinically, osteomyelitis presents in different types that vary depending on the route of infection, disease progression and patient-related factors. Osteomyelitis may be a hematogenous, vascular-insufficiency-associated, surgery-related (e.g., following joint replacements) or fracture-related infection (FRI) [2,4,6,7]. Following bacterial and/or fungal penetration into bone tissue, acute infection may develop within a few days to weeks, typically accompanied by local inflammation with fever, hyperthermia and redness and systemic responses like elevated C-reactive protein (CRP) and leukocytosis [1,8,9].

With 49 to 63%, the most frequently isolated pathogens include *Staphylococcus aureus* and coagulase-negative staphylococci (e.g., *Staphylococcus epidermidis*), among others [2,3,6,8,10,11,12,13]. Specifically, an increasing prevalence of methicillin-resistant *S. aureus* (MRSA) complicates therapy, as resistance is often associated with enhanced biofilm formation and intracellular persistence of bacteria [14,15]. In addition, *S. aureus* is able to penetrate the osteocyte-lacuno-canalicular network (OLCN), further contributing to treatment resistance [16]. Bone tissue itself generally exhibits poor perfusion, limiting oxygenation and the delivery of systemic antibiotics [1,8,10]. Bacteria frequently persist in bone tissue by forming biofilms, aided by the extracellular polymeric substance (EPS) matrix of the biofilm that confers protection against immune responses and further limits antibiotic penetration [2,3]. As a result, osteomyelitis is associated with a considerable risk of recurrence, reaching approximately 11% in FRI [4,8].

In severe or recurrent infections, surgical resection of infected bone segments frequently becomes unavoidable [4,10]. The debridement involves not only the necrotic bone but also the surrounding soft tissue and is often extensive, resulting in critical bone defects [1]. When defects exceed 2 cm in length or involve more than 50% of bone circumference, spontaneous bone regeneration is insufficient and surgical reconstruction is required [17]. Salvaging some of the resected bone for the reconstruction of the critical bone defect might represent a valuable approach as autologous reimplantation offers advantages over allografting or external fixation, including enhanced biological compatibility and preservation of native mechanical properties [18,19]. However, the complete eradication of all pathogens is an essential requirement for the re-use of the resected bone fragments. Conventional sterilization methods like heat treatment, peracetic acid/ethanol exposure or irradiation may compromise structural and biological tissue properties [20,21,22]. Gentle, tissue-preserving methods might be less effective. In particular, the disruption of mature biofilms represents a challenge. Chemical dispersal agents include for instance sodium dodecyl sulfate (SDS) as a surfactant [23] or ethylenediaminetetraacetic acid (EDTA) as an EPS matrix destabilizer [24,25]. Both agents can act synergistically with antimicrobial treatment, increasing the susceptibility of biofilm-embedded bacteria [26,27,28]. Although previous studies demonstrated efficient biofilm removal with these chemicals from surfaces, the complex microarchitecture of bone represents a substantial barrier to uniform microbial eradication by limiting chemical penetration [29]. Also, biofilm protein removal was not necessarily associated with a reduction in viable bacteria counts [29].

Therefore, the present study aimed to develop a novel bone processing protocol based on high hydrostatic pressure (HHP) treatment to synergistically enhance the inactivation of pathogens by pressure-aiding the penetration of bone with biofilm-dispersing chemicals.

Since the 1990s, HHP treatment has been used for microbial decontamination in the food industry [30]. More recently, this technology has been adapted for medical applications, specifically tissue sterilization. During HHP treatment, samples are immersed in a pressure-transmitting medium, ensuring uniform pressure distribution throughout the tissue and effective bacterial elimination [31]. In bone graft processing, HHP preserves both the mechanical integrity and the extracellular matrix properties relevant for osteo-conduction [32,33,34]. In bacteria, HHP has been shown to kill vegetative bacteria with varying efficiency dependent on growth phase, morphology, Gram classification, and strain identity [30,31,35]. Experimental data indicate that pressures of up to 600 MPa may be required to kill stationary-phase bacteria, especially Gram-positive strains such as *S. aureus* and *S. epidermidis*, both commonly associated with FRI [31,35,36].

In this in vitro study, we investigated the ability of HHP in combination with SDS and EDTA to eradicate bacterial biofilms in infected bone tissue, specifically biofilm-associated *S. aureus* and *S. epidermidis*. This combined physicochemical approach aims to meet the requirements for the safe re-use of autologous bone for reconstruction of segmental defects secondary to FRI.

## 2. Materials and Methods

### 2.1. Preparation of Bacterial Suspensions

*S. aureus* (ATCC 35556) and two strains of *S. epidermidis* (ATCC 35984 and ATCC 12228) were obtained from the American Type Culture Collection (ATCC, Manassas, VA, USA) and stored at −80 °C in glycerol. These organisms were selected, as both species are frequently associated with implant-related infections [6,10]. The inclusion of two *S. epidermidis* strains with different biofilm-forming capacities allowed us to assess whether biofilm-associated characteristics influence susceptibility to the applied decontamination approach. *S. epidermidis* strain RP62A (ATCC 35984) is a biofilm-producing, methicillin-resistant strain that was initially isolated from a patient with intravascular catheter-associated sepsis, whereas *S. epidermidis* ATCC 12228 is a non-biofilm-forming reference strain that was deposited by the U.S. Food & Drug Administration [37,38].

In general, bacteria were incubated at 37 °C and 5% CO_2_ in air (Thermo Fisher Scientific, Waltham, MA, USA) under standard laboratory incubation conditions [39]. Fresh pre-cultures were generated by inoculating single colonies into 5 mL of sterile tryptic soy broth (TSB, Sigma-Aldrich, St. Louis, MO, USA) and incubation for 16–18 h. Pre-cultures were used to inoculate main cultures of 20 mL TSB in a 1:20 ratio. If not stated otherwise, stationary phase bacteria were harvested after 22–24 h of cultivation.

### 2.2. Culture-Based Assessment of Viable Bacteria Numbers

For all experiments, the treated samples consisted of bacterial suspensions or biofilm-associated bacteria exposed to the respective experimental condition, i.e., SDS/EDTA (SDS: Carl Roth GmbH + Co. KG, Karlsruhe, Germany; EDTA: Sigma-Aldrich, St. Louis, MO, USA) of different concentrations in 0.9% saline with or without application of HHP. In general, controls consisted of samples incubated in 0.9% saline. All samples were processed under identical conditions for colony forming units (CFU) determination.

For determination of CFU/mL, bacterial suspensions were serially diluted in 0.9% saline, plated on TSB agar (Sigma-Aldrich, St. Louis, MO, USA) and incubated at 37 °C and 5% CO_2_ in air for 24 h.

Differences in viable cell counts were expressed as logarithmic reduction factors (logRF) based on Equation (1). However, when N = 0, i.e., no colonies were detected, the calculation of the logRF is not possible. Thus, in cases where no colonies were detected, by definition the presence of a single colony was presumed which corresponded to a value of 56 CFU/mL in the log reduction calculations, based on the detection limit of the assay according to the plated volume (50 µL) and the resuspension volume of 2.8 mL in the cryogenic vials. As logRF values are calculated relative to the CFU of the 0.9% saline control (Equation (1)), the resulting maximum achievable log reduction (logRF_max_) varies between experimental setups depending on the number of viable bacteria in the control. The logRF_max_ is indicated in the respective figures and tables. Log reductions are displayed as negative values (e.g., −5 corresponds to a reduction by 5 log_10_, i.e., viable cells were reduced by a factor of 10^5^).

(1) $$\mathrm{logRF}\;=\;\log\frac{N}{N_{0}}$$

RF is the reduction factor; N is the viable count after treatment [CFU/mL]; and N_0_ is the viable count w/o treatment (control) [CFU/mL].

### 2.3. Evaluation of Effects of Chemical Dispersal Agents in Bacterial Suspensions

#### 2.3.1. Checkerboard Assay

To test the minimum inhibitory concentration of a combination of SDS and EDTA, a checkerboard assay was performed. Here, different concentrations ranging from 0.0008% to 0.05% SDS and 0.02 mM to 5 mM EDTA in 0.9% saline were tested for their ability to inhibit *S. aureus* growth alone and in combination. 10 µL of the substance combinations were mixed with 90 µL of 5 × 10^5^ CFU/mL bacterial suspension in cation-adjusted Mueller-Hinton broth (CAMHB, Sigma-Aldrich, St. Louis, MO, USA) per well in a 96-well plate. Negative controls contained 10 µL of the respective substance combinations and 90 µL of sterile CAMHB, positive controls were bacterial suspensions without substance. After 24 h of incubation, the optical density at 600 nm (OD_600_) was quantified using a FLUOstar OMEGA microplate reader (BMG LABTECH, Ortenberg, Germany). Cut-off for visual growth was set to a total increase in OD_600_ of less than 0.05. Here, the minimum inhibitory concentration of the additive combination (MIC_comb_) was defined as combination of substance containing the lowest possible concentrations that resulted in growth inhibition in all three independent experiments. Fractional inhibitory concentration (FIC) indices were calculated using Equation (2):

(2) $$\mathrm{FIC}\;\mathrm{index}\;=\;\frac{{\mathrm{MIC}}_{\mathrm{SDS}\;\mathrm{comb}}}{{\mathrm{MIC}}_{\mathrm{SDS}}}\;+\;\frac{{\mathrm{MIC}}_{\mathrm{EDTA}\;\mathrm{comb}}}{{\mathrm{MIC}}_{\mathrm{EDTA}}}$$

FIC, fractional inhibitory concentration; MIC_SDS comb_, minimum inhibitory concentration of SDS in combination with EDTA; MIC_SDS_, minimum inhibitory concentration of SDS alone; MIC_EDTA comb_, minimum inhibitory concentration of EDTA in combination with SDS; MIC_EDTA,_ minimum inhibitory concentration of EDTA alone.

Based on the MIC_comb_ determined in *S. aureus*, a checkerboard reference concentration (CRC) was defined and used within the following experiments (Table 1).

#### 2.3.2. Time–Kill Assay

Time–kill assays were conducted to assess the kinetics of dose combinations (Table 1) identified in the checkerboard assay.

For each condition and sampling time point, individual samples were prepared. 1 mL of bacterial suspension was harvested by centrifugation (3000× *g*, 5 min) in a 2 mL cryogenic tube. Supernatants were discarded and cryogenic tubes were filled with SDS/EDTA medium (Table 1) air-bubble free, corresponding to the experimental setup of Section 2.4. The sealed tubes were incubated at 4 °C and plated after defined exposure times (15 min, 1 h, 2 h and 3 h) to evaluate changes in viable cell counts, reported as logRF (Equation (1)).

### 2.4. Evaluation of Effects of HHP Plus SDS and EDTA in Planktonic Bacteria

For the treatment of bacterial suspensions, 1 mL of a stationary-phase culture was transferred to a 2 mL cryogenic tube (Thermo Fisher Scientific, Waltham, MA, USA). After centrifugation (3000× *g*, 5 min), the supernatant was discarded and the pelleted bacteria were resuspended in the pressure-transmitting medium that contained SDS and EDTA in the concentrations as detailed in Table 1. The tube was filled completely with the medium to ensure it was free of air bubbles. The bacteria were immersed in the pressure-transmitting medium with or without additives at 4 °C for a total incubation period of 3 h. This exposure time was kept constant to control for potential time-dependent effects of the additives. HHP treatment was performed within this 3 h incubation period to ensure standardized exposure conditions for all samples.

For HHP treatment, the sealed cryogenic tubes were placed in 15 mL Falcon Tubes filled with water, which were then placed in the pressurization chamber of the HHP device (HDR-100, RECORD GmbH, Koenigsee, Germany), filled with a 1:1 mixture of glycol and water. The pressure was increased and decreased at a rate of approximately 2.5 MPa/s. The HHP treatment was performed either at 250 MPa or at 350 MPa for 20 min at 10 °C. During this time interval the control samples (0.9% saline, 0 MPa) were kept continuously in the respective medium in the cryotubes at 4 °C. Subsequently, viable bacteria were determined as described in Section 2.2, using the untreated samples in 0.9% saline as controls.

### 2.5. Evaluation of Effects of HHP Plus SDS and EDTA in Biofilm-Infected Bone Samples

To test the physicochemical approach in a 3D bone environment, biofilms were established in trabecular bone cylinders. The human trabecular bone cylinders were obtained from femoral heads from patients undergoing total hip replacement, under informed consent and corresponding ethics approval (local Ethics Committee of the University of Rostock; registration number A 2010-0010). Femoral heads were stored at −20 °C. After gentle thawing, samples were sawn into cancellous disks of approximately 10 mm in height from which cylinders with a diameter of 6 mm were obtained using a trepan drill (Ustomed Instrumente, Tuttlingen, Germany) under continuous cooling with phosphate-buffered saline (PBS). In order to ensure sterility in the biofilm assays, bone samples were treated with a graded ethanol series, using concentrations of 50%, 70%, 80%, 90%, and 96% ethanol for 10 min each, followed by washing for 2 × 10 min in PBS before being air-dried under a laminar flow for 30 min to ensure the absence of ethanol residues. Due to the clinical origin of the samples, the bone cylinders represented heterogeneous human trabecular bone specimens with inherent donor- and location-dependent variability. Samples were therefore considered as independent biological specimens for the evaluation of treatment effects.

To establish bacterial biofilms, bone samples were incubated in 2 mL TSB inoculated with 10 µL of a pre-culture of *S. aureus* (ATCC 35556) or *S. epidermidis* (ATCC 35984), yielding a final inoculum of approximately 4 × 10^6^ CFU/mL. Bones were cultivated in a 24-well plate for 72 h, with medium being replaced every 24 h. Biofilm-infected bone cylinders were transferred to 2 mL cryogenic tubes and treated in the same way as planktonic bacteria (Section 2.4). Briefly, the biofilm-infected bone cylinders were incubated in the pressure-transmitting medium with or without additives at 4 °C for 3 h. During this chemical treatment, bone cylinders were subjected to either 250 MPa or 350 MPa HHP for 20 min. Treatment time and pressure levels were based on previous experiments that were conducted to optimize bacterial killing while preserving the structure of the extracellular matrix proteins in bone [32,35].

To quantify viable bacteria after HHP treatment, ultrasound at 45 kHz for 5 min (VWR USC 200T, Leuven, Belgium) was applied to all bone cylinders to detach surface-bound bacteria and disperse aggregates for plating at defined concentrations. Afterwards, CFUs were quantified as described in Section 2.2.

### 2.6. Scanning Electron Microscopy (SEM)

For SEM imaging, bone disks of 1–2 mm in height and 6 mm in diameter were sterilized and defatted by a graded ethanol series, and biofilms of *S. aureus* ATCC 35556 or *S. epidermidis* ATCC 35984 were prepared as described in Section 2.5. HHP was applied at 350 MPa using either 0.9% saline or 0.05% SDS/5 mM EDTA in 0.9% saline, compared to untreated controls. These treatment conditions were selected to represent the highest pressure level and additive concentration tested, which showed the strongest antimicrobial effects in the biofilm experiments. Samples were fixed in 0.1 M sodium phosphate fixation buffer (pH = 7.3) containing 1% paraformaldehyde and 2.5% glutaraldehyde, and stored at 4 °C.

After fixation, the bone disks were washed twice with sodium phosphate buffer and dehydrated in an ascending acetone series (30%, 50%, 70%, 90%, 100%). The samples were then critical point dried using CO_2_ (Emitech K850/Quorum Technologies Ltd., East Sussex, UK). After mounting on SEM specimen holders using carbon adhesive tape (Plano, Wetzlar, Germany), bone disks were coated with a carbon layer (15 nm) under vacuum (CCU 010 HV-Coating Unit, Co. Safematic GmbH, Zizers, Switzerland) to establish surface conductivity. Electron microscopy was performed using a field emission scanning electron microscope (Zeiss Merlin VP compact, Carl Zeiss, Oberkochen, Germany; applied detectors: high efficiency secondary electron detector (HE-SE) and InlensDuo detector; acceleration voltage: 5.0 kV; working distance: 2.9–3.8 mm).

### 2.7. Graphical Illustration and Statistical Analyses

GraphPad Prism 9.0 (GraphPad Software, Boston, MA, USA) was used for graphical illustration and statistical analyses. Logarithmic reduction factors are displayed as mean values ± standard deviation (SD). Statistical significance was assessed using two-way ANOVA with Dunnett’s multiple comparisons test to evaluate the effects of the two experimental factors (i.e., time and additive concentration or pressure level and additive concentration) as well as their interaction. In general, *p*-values < 0.05 were considered statistically significant.

## 3. Results

### 3.1. Additive Dose Determination

Minimum inhibitory concentrations (MICs) of SDS and EDTA alone and in combination were determined using a checkerboard assay to assess additive effects and define a checkerboard reference concentration (CRC) for subsequent experiments. Table 2 summarizes the results of three independent experiments with *S. aureus* ATCC 35556.

FIC indices between 0.5 and 1 were considered indicative of an additive effect [40]. As displayed in Table 2, the combination of SDS and EDTA showed an average FIC index of 0.70 ± 0.09, suggesting an additive effect of SDS and EDTA. For subsequent experiments, the lowest effective combination of both substances that led to complete growth inhibition (0.0063% SDS; 0.63 mM EDTA) was selected and defined as MIC_comb_, equaling 1× CRC in all following experiments.

### 3.2. Time–Kill Kinetics of Bacterial Killing by SDS and EDTA

**Figure 1:**
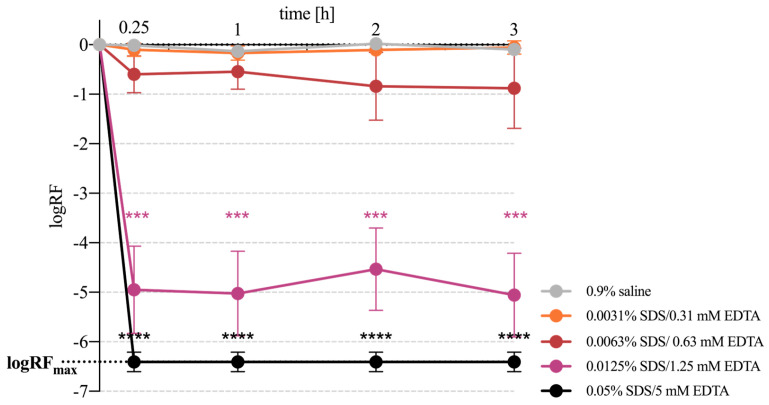
Time–kill kinetics of stationary phase *S. aureus* ATCC 35556 in response to treatment with different concentrations of SDS and EDTA. Cultures were incubated for 22–24 h in TSB medium. 1 mL was harvested by centrifugation and resuspended in approximately 2.8 mL of treatment medium in a 2 mL cryogenic tube. In total, 0.9% saline (grey, control) was supplemented with additive combinations ranging from ½× CRC to 8× CRC: orange, 0.0031% SDS/0.31 mM EDTA; red, 0.0063% SDS/0.63 mM EDTA; pink, 0.0125% SDS/1.25 mM EDTA; black, 0.05% SDS/5 mM EDTA. After defined exposure times, cells were plated on TSB agar. Bacterial inactivation is expressed as logarithmic reduction, defined as logRF = log(N/N_0_). Thus, a logRF of −5 corresponds to a reduction of 5 log_10_. Data represent mean values ± standard deviation (n = 5). Dotted line: maximum achievable log reduction, logRF_max_. Statistical significance was assessed using a repeated measures two-way ANOVA followed by Dunnett’s multiple comparisons test: *** *p* < 0.001; **** *p* < 0.0001.

To assess the time-dependent kinetics of bacterial killing, SDS and EDTA were applied at concentrations half to eight times the identified CRC (0.0063% SDS; 0.63 mM EDTA) for up to 3 h, as shown in Figure 1. Corresponding CFU values are depicted in Table A1.

Killing of bacteria did not seem to follow a clear time-dependent pattern as plateaus were reached after 15 min of chemical treatment with no statistical differences between later time points. At 0.0031% SDS/0.31 mM EDTA and 0.0063% SDS/0.63 mM EDTA, no significant decrease in viable counts was observed. 0.0063% SDS/0.63 mM EDTA reduced viable counts by approximately 1 log_10_, while 0.0125% SDS/1.25 mM EDTA resulted in a significant reduction of 5 log_10_. At 0.05% SDS/5 mM EDTA, no growth was detectable after 15 min of treatment in all five replicates (*p* = 0.0108).

### 3.3. Combined Effect of HHP and Additive Medium in Planktonic Staphylococci

For the combined treatment with HHP and SDS/EDTA, pressure levels of 250 MPa and 350 MPa and concentrations of up to 0.05% SDS/5 mM EDTA were tested. *S. aureus* ATCC 35556 and two different strains of *S. epidermidis* (ATCC 35984 and ATCC 12228) were selected for the experiments.

Across all tested strains and conditions, additive concentration and pressure level had a significant impact on the reduction in viable bacteria (*p*_all_ < 0.0001). Results are summarized in Table 3. Corresponding CFU values are depicted in Table A2. The reported post hoc analyses concentrate on the effect that increasing the HHP levels had on the log_10_ reduction in the respective additive concentration of SDS/EDTA.

In all planktonic bacteria, 0.0125% SDS/1.25 mM EDTA led to decrease in viability independent of the pressure level that exceeded 5 log_10_ in *S. aureus* and *S. epidermidis* ATCC 12228. Exposure of bacteria in 0.05% SDS/5 mM EDTA sterilized the bacterial suspensions and therefore reached the logRF_max_.

In planktonic suspensions of *S. aureus*, HHP treatment in 0.9% saline alone resulted in limited inactivation, whereas increasing SDS/EDTA concentrations led to a concentration-dependent increase in efficiency. The strongest increase in efficiency vs. the non-pressurized control was observed for HHP treatment with 0.0063% SDS/0.63 mM EDTA, where HHP at both 250 MPa and 350 MPa enhanced the viable count reduction from approximately 2 log_10_ to >5 log_10_.

In general, *S. epidermidis* showed greater susceptibility to HHP than *S. aureus*. *S. epidermidis* ATCC 35984 showed high sensitivity towards HHP treatment, with significant reduction already reached at 250 MPa and with 0.9% saline. This substantial reduction was further enhanced by increasing SDS/EDTA concentrations, reaching its maximum with >0.0125% SDS/1.25 mM EDTA at 250 MPa and at >0.0063% SDS/0.63 mM EDTA at 350 MPa. *S. epidermidis* ATCC 12228 was also susceptible to HHP, with no viable bacteria observed at 350 MPa regardless of additive concentration. At 250 MPa, no CFUs were detected at ≥0.0063% SDS/0.63 mM EDTA, while lower concentrations resulted in reduced but still substantial bacterial killing.

### 3.4. Combined Effect of HHP and Additive Medium in Biofilm-Associated Staphylococci

*S. aureus* ATCC 35556 and *S. epidermidis* ATCC 35984 were cultivated on human trabecular bone cylinders to investigate effects on biofilm formation. Across both tested strains and all conditions, additive concentration and pressure level had a significant impact on the reduction in viable bacteria (*p*_all_ < 0.0001). The only exception was observed for *S. aureus* biofilms, where the impact of pressure was comparatively weaker, despite remaining statistically significant (*p* = 0.0004). Results are summarized in Table 4. Corresponding CFU values are depicted in Table A3.

*S. aureus* biofilm showed pronounced resistance against HHP treatment using 0.9% saline, leading to a maximum reduction of 0.66 ± 0.19 log_10_ at 350 MPa. While 0.0125% SDS/1.25 mM EDTA itself did not have a significant impact on viability, the combination with HHP enhanced inactivation to approximately 2 log_10_ at 350 MPa. 0.05% SDS/5 mM EDTA had a significant impact on bacterial reduction with increasing pressure levels yielding even stronger effects. In the treatment of *S. epidermidis* biofilm, the pressure itself had a stronger impact on the reduction than in *S. aureus*. The addition of SDS and EDTA (0.0125% SDS/1.25 mM EDTA) elevated the reduction to approximately 2 log_10_ for 250 MPa and 5 log_10_ for the treatment at 350 MPa. Treatment using 0.05% SDS/5 mM EDTA led to no detectable CFU in all three samples treated at 250 MPa and 350 MPa.

### 3.5. Morphological Evaluation of Bone-Associated Bacterial Biofilms After HHP Treatment Combined with Additives

**Figure 2:**
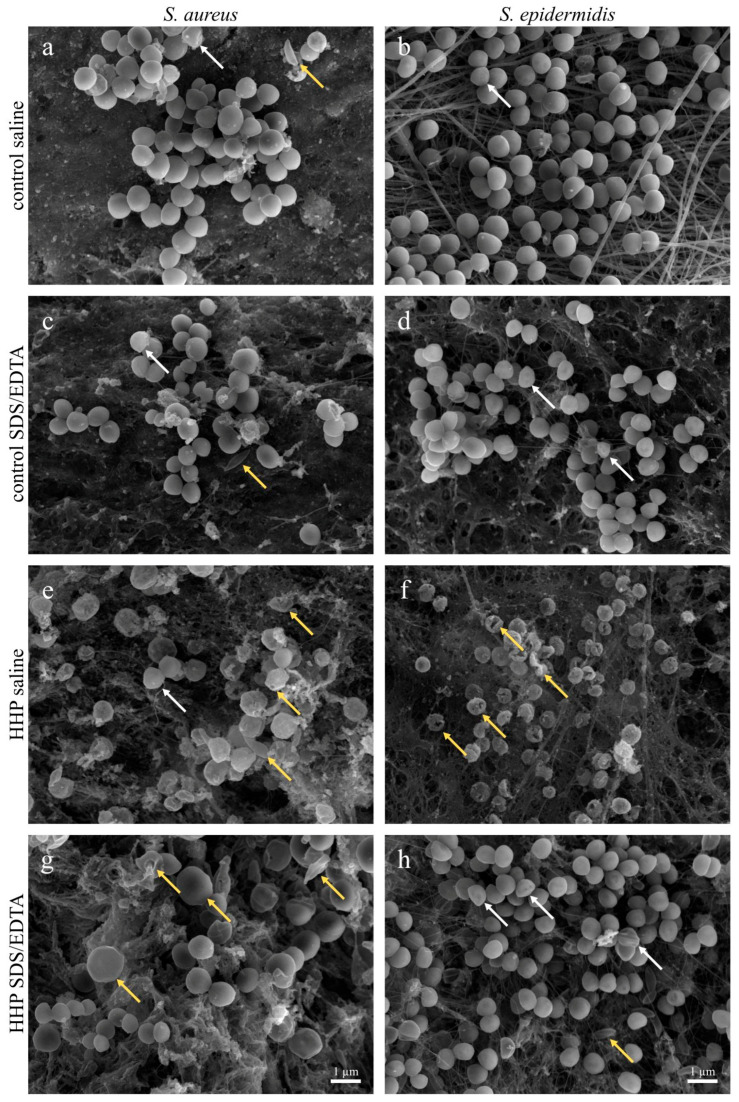
Morphological changes in biofilms of *Staphylococcus* spp. on human trabecular bone towards HHP treatment. Human trabecular bone disks were cultivated with *S. aureus* ATCC 35556 (**a**,**c**,**e**,**g**) or *S. epidermidis* ATCC 35984 (**b**,**d**,**f**,**h**) for 72 h. Bone disks immersed in 0.9% saline (**a**,**b**,**e**,**f**) or 0.05% SDS/5 mM EDTA (**c**,**d**,**g**,**h**) were treated with HHP at 350 MPa (**e**–**h**) or remained untreated (**a**–**d**). After treatment, bone samples were processed for scanning electron microscopy. Images were acquired by mixing signals of HE-SE and InLens in a 1:1 ratio. Scale bars represent 1 µm. Bacterial cells showed various reactions towards treatment, including irregular cell wall morphology (white arrows) and further structural damage characterized by local envelope disruption, swelling and collapse of bacteria (yellow arrows). The two morphological categories are interpreted as different degrees of cell envelope damage. EDTA, ethylenediaminetetraacetic acid; HHP, high hydrostatic pressure; SDS, sodium dodecyl sulfate.

Human trabecular bone disks were treated with HHP in pressure-transmitting media supplemented with 0.05% SDS and 5 mM EDTA compared to 0.9% saline to evaluate morphological changes in biofilm induced by HHP treatment with or without additives (Figure 2).

Scanning electron microscopy of staphylococcal biofilms on trabecular bone showed bacterial cells embedded in a matrix interconnecting neighboring bacteria through fine filamentous structures, which likely represent extracellular matrix components such as polysaccharides or extracellular DNA. Clear morphological alterations depending on treatment conditions were observed as presented in Figure 2. The observed changes comprised different degrees of bacterial cell envelope damage, which were grouped into two morphological categories representing increasing severity (white and yellow arrows).

In untreated control samples (Figure 2a,b), bacteria appeared largely intact and smooth, showing only minor surface irregularities. Exposure to SDS and EDTA without application of pressure (Figure 2c,d) led to deformation and surface irregularities (white arrows) and occasional collapse of bacterial envelopes (yellow arrows) that was comparably pronounced in both *S. aureus* (Figure 2c) and *S. epidermidis* (Figure 2d).

HHP treatment at 350 MPa in 0.9% saline (Figure 2e,f) resulted in noticeable structural changes, including surface roughening (white arrows). Bacteria within *S. epidermidis* biofilms (Figure 2f) showed severe cell envelope perforation and collapse (yellow arrows) that seemed more pronounced than in *S. aureus* (Figure 2e). Distinct and heterogenous morphological alterations were observed in samples exposed to SDS/EDTA and HHP at 350 MPa (Figure 2g,h). Collapsed and locally perforated (yellow arrows) as well as irregularly shaped (white arrows) cells were observed. In *S. aureus* biofilms treated at 350 MPa with SDS/EDTA, some bacteria seemed swollen (yellow arrows, Figure 2g).

Notably, the extent of morphological damage varied within and between treatment conditions, reflecting the heterogeneity of biofilm-associated and biofilm-embedded bacteria. Overall, the SEM images showed a progression from mostly intact cells in untreated biofilm to increasingly heterogeneous and structurally altered morphologies following additive and pressure treatment.

## 4. Discussion

Osteomyelitis remains among the most persistent challenges in orthopedic and trauma surgery, particularly due to its high recurrence rate [1,2,4,6]. Successful treatment requires complete pathogen eradication while preserving bone integrity. In view of these challenges, strategies that enable the sterilization and reuse of autologous bone segments may substantially advance reconstructive treatment concepts by combining microbial safety with optimal anatomical, biological, and immunological compatibility.

Both *S. aureus* and *S. epidermidis* represent clinically relevant pathogens in FRI [6,13]. Their pronounced capacity to form biofilms and persist within bone microstructures markedly reduces their susceptibility to conventional therapy [1,2,10,16,41]. To overcome bacterial defense mechanisms such as antibiotic resistance, physicochemical decontamination methods, including HHP, have gained increasing attention. However, Gram-positive bacteria such as *S. aureus* exhibit intrinsic pressure resistance, limiting its standalone efficacy [31,35,36]. Previous studies have shown that HHP combined with additives such as lysozyme and EDTA enhances bacterial killing by promoting membrane destabilization [42,43,44]. Based on these considerations, we investigated if combined SDS/EDTA treatment could sensitize staphylococci in suspension and bone-associated biofilms to subsequent HHP exposure [26,27,45,46,47].

Previous studies on detergent-based tissue processing have highlighted that SDS and EDTA exhibit concentration-dependent effects on both cellular removal and extracellular matrix preservation, emphasizing the need to balance antimicrobial efficacy with structural preservation of biological tissues [48]. Checkerboard analysis demonstrated additive activity between SDS and EDTA, reflected by a fractional inhibitory concentration (FIC) index of 0.70 ± 0.09, allowing the reduction in the additive concentrations for subsequent HHP experiments when both substances were combined. The determined concentrations of 0.0063% SDS and 0.63 mM EDTA are consistent with previously reported minimum inhibitory concentrations (MICs) for *S. aureus* of 0.004% SDS [27] and 0.2–2.4 mM EDTA [49,50]. In line with earlier reports, EDTA alone predominantly exerted growth-inhibitory rather than bactericidal effects [49,50], whereas SDS induced a moderate reduction of approximately 1 log_10_ in viable counts after 3 h at concentrations of 0.02% to 0.04% [51].

The enhanced additive activity of SDS and EDTA can be attributed to complementary mechanisms of action. SDS integrates into the lipid bilayer, disrupting its integrity and denaturing surface proteins, whereas EDTA chelates cell-wall-stabilizing divalent cations, such as Ca^2+^ and Mg^2+^ [26,27,45,46,47,52,53]. This dual mode of action promotes membrane destabilization and permeabilization, ultimately resulting in the bactericidal effects observed in the suspension assays. No pronounced time-dependent effect was observed for the killing of *S. aureus* treated with SDS/EDTA, suggesting a rapid onset of membrane disruption rather than cumulative damage.

While SDS and EDTA have been widely studied in tissue processing, their effects are highly concentration-dependent, ranging from mild membrane permeabilization to more pronounced disruption of extracellular structures [48]. This underscores the rationale for using reduced additive concentrations in combination with HHP to minimize potential damage while maintaining antimicrobial efficacy.

Having established the effects of SDS/EDTA alone, we next assessed the susceptibility of bacterial suspensions to HHP. HHP-mediated inactivation was evaluated for *S. aureus* ATCC 35556 and two *S. epidermidis* strains (ATCC 35984 and ATCC 12228). Among the tested organisms, *S. aureus* exhibited the highest tolerance to pressure, consistent with its well-documented resistance to pressure-induced inactivation [36,43,54,55]. In contrast, both *S. epidermidis* strains displayed markedly higher susceptibility. This differential response underscores the pronounced inter-species variability in bacterial susceptibility to HHP [31,56].

Although *S. aureus* and *S. epidermidis* share similar morphology and Gram characteristics, differences in cell wall architecture and stress adaptation systems likely account for their differential response to HHP. For instance, *S. aureus* possesses membrane-modifying systems, a robust sigma factor SigB-mediated general stress response, and adaptive regulation of the peptidoglycan homeostasis [57,58]. These mechanisms improve membrane and cell wall stability as well as protein turnover, which are key cellular targets of HHP [31,59,60]. In contrast, *S. epidermidis* exhibits lower intrinsic virulence and relies predominantly on biofilm formation as its principal strategy [10,15]. The two *S. epidermidis* strains included in this study differed in their biofilm-forming capacity, with ATCC 35984 representing a biofilm-forming strain and ATCC 12228 lacking this phenotype. However, both strains showed high susceptibility to HHP, suggesting that the general capacity for biofilm formation alone was not a dominant determinant of pressure-mediated inactivation under the tested conditions.

At higher additive concentrations (>0.0125% SDS/1.25 mM EDTA), substantial bacterial reduction was achieved without HHP. However, HHP markedly enhanced killing at sublethal concentrations, indicating an additive effect of chemical and physiochemical treatment. Since even small numbers of residual bacteria may trigger reinfection, the additional reduction achieved by HHP may improve the overall safety margin of the decontamination process [61]. In *S. epidermidis*, the additional benefit of SDS and EDTA was comparatively modest; nevertheless, their inclusion remains advantageous for reliably achieving decontamination levels ≥5 log_10_ as defined by DIN EN standards [62,63].

Mechanistically, SDS/EDTA and HHP target overlapping cellular structures via distinct physicochemical pathways resulting in enhanced combined efficiency. HHP primarily disrupts non-covalent interactions within macromolecules, causing reversible protein unfolding at around 200 MPa to 300 MPa and irreversible denaturation above 400 MPa [31,59,64]. These alterations impair enzymatic activity, leading to a collapse of energy metabolism and ultimately abolishing reproductive capacity [31,59,64]. Concurrently, HHP affects membrane fluidity and permeability, promoting nutrient leakage and cell death [35,65,66]. Under these conditions, mild chemical destabilization by SDS and EDTA is likely to be amplified, as HHP-induced changes in the membrane facilitate additive penetration and cation chelation. In turn, the loss of protein stability and membrane integrity may sensitize bacteria to further HHP-induced cell death. This interplay plausibly explains substantially higher inactivation rates achieved by the combined treatment compared with either modality alone.

As biofilm formation represents a major determinant of treatment failure in osteomyelitis, bacterial susceptibility under bone-associated biofilm conditions was systematically examined [14,15]. After 72 h of incubation on trabecular bone cylinders, both *S. aureus* ATCC 35556 and *S. epidermidis* ATCC 35984 formed biofilms typically characterized by aggregates of EPS, including polysaccharides and extracellular DNA, which markedly restrict the diffusion of additives [45,67]. Staphylococcal biofilms exhibited a markedly higher resistance toward HHP and chemical additives compared to planktonic bacteria. Within this study, *S. aureus* biofilms demonstrated only minor sensitivity to HHP alone, while additives markedly increased inactivation in a concentration-dependent manner. *S. epidermidis* biofilms were more susceptible to HHP, resulting in no detectable CFU at 250 MPa. Biofilm-associated cells experience environmental stress, including nutrient limitation, ATP depletion, and low oxygen availability. These conditions lead to decreased metabolic activity, altered cell wall architecture and increased efflux activity, contributing to the elevated resistance of biofilms to antibiotics and likely also to their physicochemical resistance [10,15,68,69,70,71]. Although biofilm-dispersed cells are known to transiently retain stress-adaptive phenotypes after detachment, the extent to which these mechanisms influenced HHP susceptibility in the present study remains to be clarified [72]. Moreover, the porous bone microstructure may act as a physical barrier, limiting uniform pressure transmission and chemical penetration [73]. Within bacterial biofilms, SDS and EDTA likely act not only on bacterial membranes but also on the extracellular matrix itself. By solubilizing matrix-associated proteins, these agents may loosen the biofilm structure, while simultaneously damaging the embedded cells. This combined matrix impairment and membrane destabilization plausibly explains the strong HHP-mediated reduction in viability observed in the presence of SDS and EDTA [26,27,45,46,47]. Therefore, chemical weakening of biofilm matrix and cell envelope seems to be critical for enhancing HHP efficacy in this model [45,67].

To correlate viability data with structural alterations, scanning electron microscopy was performed. SEM images confirmed the presence of a biofilm layer attached to the trabecular bone surface. Fine filamentous connections between bacteria and the surrounding tissue likely represent EPS fibers interwoven with extracellular DNA and host-derived proteins [10,45,67]. Images revealed morphological alterations in staphylococcal biofilms depending on treatment conditions. Exposure to HHP and SDS/ETDA caused pronounced but morphologically heterogenous structural damage in *S. aureus*, with cells exhibiting uneven cell walls and appearing perforated or collapsed. In *S. epidermidis* biofilms, pronounced cell wall disruption was evident already under milder HHP conditions in saline alone, consistent with the higher pressure sensitivity observed in viability assays. Occasional cell swelling as observed in *S. aureus* biofilms may reflect osmotic imbalance due to membrane permeabilization representing a further expression of structural cell envelope damage [42,65,66]. Similarly, pressure-induced lesions, including membrane ruptures and leakage of intracellular content, have previously been described as hallmarks of bacterial killing under hydrostatic compression [60,65,66]. Despite the extensive structural damage observed by SEM, the majority of bacteria retained their basic cellular morphology, suggesting that cell wall remnants and residual envelopes persist even after complete loss of viability as determined in the quantitative assays. This dissociation between morphological appearance and viability corresponds with earlier studies showing that high hydrostatic pressure primarily disrupts membrane integrity and denatures essential proteins, but does not necessarily lead to full physical disintegration of the bacterial cell [42,43,55,65,66].

While the present study provides detailed insights into the extent of inactivation and structural bacterial damage under in vitro conditions, the situation in vivo is inherently more complex. The current model does not fully reflect the heterogeneity of clinical osteomyelitis, including polymicrobial infections, host-specific factors, inflammatory responses, and the presence of mature biofilms developing over longer time periods. Specifically, the presented model was limited to single-species biofilms of 72 h. Although this approach enables standardized comparison of treatment effects, clinically relevant biofilms may exhibit increased heterogenicity as well as resistance [74]. Depending on the affected bone, 6–57% of osteomyelitis cases are mixed infections [75,76]. Future studies should therefore utilize models that more closely reflect clinical situations, including polymicrobial biofilms and longer incubation times.

In addition, the present study was performed using well-characterized ATCC reference strains, enabling standardized comparison of treatment effects but not fully reflecting the diversity of clinical isolates, only including a methicillin-resistant *S. epidermidis* strain, but no MRSA or multidrug-resistant coagulase-negative staphylococci [15]. Furthermore, intracellular bacterial persistence as well as the presence of protected microenvironments within infected bone tissue that may influence treatment efficiency will need to be addressed in further studies.

Beyond bacterial viability, the biological consequences of bone reimplantation after ex vivo decontamination remain incompletely understood. From an immunological perspective, the persistence of non-viable bacteria may still hold relevance, as residual cell wall fragments like peptidoglycan, teichoic and lipoteichoic acids can act as pathogen-associated molecular patterns (PAMPs) that trigger inflammatory responses. Furthermore, the presence of viable but non-culturable bacteria (VBNC) with full immunological potential is conceivable [77,78,79,80]. This highlights a general limitation of culture-based readouts, which may underestimate residual bacterial activity. Future studies may benefit from complementary molecular and viability-based approaches, such as PCR-based assays, transcriptomic analyses, or fluorescence-based viability staining, to better characterize residual bacterial burden and stress-adapted phenotypes following treatment [81].

Moreover, potential residues of SDS and EDTA on bone surfaces require careful evaluation. Although the concentrations used in this study are far below those typically applied for decalcification (10–14% EDTA) [82,83,84] or tissue decellularization protocols using SDS (0.5–1%) [48,85], even minor residual amounts may affect osteoblasts. Depending on the cell type, EDTA and SDS have been shown to be cytotoxic at concentrations as low as 0.05 mM and 0.0001%, respectively [86,87,88,89,90]. It is therefore sensible to keep the initial concentrations of these chemicals as low as possible by using them in combination with HHP. To further mitigate these risks, future work should establish standardized washing protocols to remove chemical and biological residues from the porous trabecular network. Such protocols should be validated using quantitative residue analytics, for example HPLC-based detection, mass spectrometry approaches or novel non-destructive methods of residue quantification [89]. Importantly, previous studies have indicated that SDS-related alterations in extracellular matrix composition and structure may affect cellular repopulation independent of residual detergent concentration, highlighting the need to assess both chemical clearance and tissue-level effects [91]. Those methods should be combined with functional assessment of osteoblast viability, osteogenic differentiation, extracellular matrix formation, and potential inflammatory responses. These analyses will be essential to determine whether chemically treated bone retains sufficient biological compatibility for subsequent osseointegration. Therefore, the current findings should be interpreted as demonstrating enhanced microbial decontamination under controlled experimental conditions rather than proof of clinical sterility or immediate applicability for bone reimplantation.

In a clinical setting, surgical management in FRI and osteomyelitis infections always includes systemic antibiotic therapy apart from surgical debridement and extensive irrigation [1,92]. Systemic antibiotic therapy supported by host immune clearance might thus clear the residual bacterial burden in the autografts. Consequently, a ≥5 log_10_ reduction achieved ex vivo may represent a clinically meaningful reduction in the infectious load even if sterility (SAL, sterility assurance level = 10^−6^) is not yet reached [62,63]. From a translational perspective, this residual bacterial reduction must be interpreted in the context of subsequent antibiotic therapy and host immune control rather than as an isolated end-point of decontamination efficacy.

## 5. Conclusions

In conclusion, this study demonstrates that low-dose SDS/EDTA in combination with HHP enables effective killing of both planktonic and bone-associated staphylococci. Notably, neither HHP nor SDS/EDTA alone provides an optimal balance between antimicrobial efficacy and tissue preservation; in combination, however, both pressure and additive concentrations can be reduced while maintaining effective bacterial inactivation.

While the achieved levels of bacterial reduction are substantial, current regulatory requirements for complete sterilization (e.g., sterility assurance level, SAL 10^−6^) are not yet fulfilled, indicating that further optimization is required prior to clinical application. Although culture-based methods remain the regulatory gold standard for sterility assessment, emerging rapid and risk-based microbial testing approaches may provide complementary tools for future evaluation of complex regenerative manufacturing processes [81].

From a translational perspective, this HHP-based approach might be integrated into a two-stage surgical workflow in which infected bone is resected, sterilized ex vivo, and reimplanted after verification of sufficient decontamination. This strategy aligns with current treatment concepts for chronic osteomyelitis while potentially reducing the risk of donor-site morbidity and improving anatomical reconstruction. With further optimization of additive protocols, verification of immunological safety, and confirming biomechanical stability, this approach may ultimately contribute to safe and effective autologous reconstruction strategies for FRI.

## Figures and Tables

**Table 1:** Concentrations of SDS and EDTA in 0.9% saline tested in the time–kill assay.

Medium	SDS [%]	EDTA [mM]
Control (0.9% saline)	0	0
½× CRC	0.0031	0.31
1× CRC	0.0063	0.63
2× CRC	0.0125	1.25
8× CRC	0.05	5

CRC, checkerboard reference concentration; EDTA, ethylenediaminetetraacetic acid; SDS, sodium dodecyl sulfate.

**Table 2:** Minimum inhibitory concentrations (MICs) of SDS and EDTA alone and in combination against *S. aureus* ATCC 35556, including fractional inhibitory concentration (FIC) indices. Bacteria were cultivated in Tryptic Soy Broth (TSB) medium at 37 °C and 5% CO_2_ in air for 24 h. FIC indices were calculated based on Equation (2).

Replicate	MIC SDS [%]	MIC EDTA [mM]	MIC_comb_ SDS [%]	MIC_comb_ EDTA [mM]	FIC Index
1	0.0125	2.5	0.0063	0.16	0.57
2	0.0125	2.5	0.0063	0.63	0.76
3	0.0125	2.5	0.0063	0.63	0.76
mean ± SD	0.0125	2.5	0.0063	0.47 ± 0.22	0.70 ± 0.09

EDTA, ethylenediaminetetraacetic acid; FIC, fractional inhibitory concentration; MIC, minimum inhibitory concentration; MIC_comb,_ minimum inhibitory concentration of the additive combination; SD, standard deviation; SDS, sodium dodecyl sulfate.

**Table 3:** Detailed log_10_ reductions and statistical comparisons for planktonic *S. aureus* and *S. epidermidis* under varying SDS/EDTA concentrations and pressure levels. Bold log_10_ reduction values: maximum achievable log reduction, logRF_max_; italic *p*-values: statistically significant vs. non-pressurized controls.

Sample Organism (ATCC)	Pressure-Transmitting Medium	Pressure Level [MPa]	log_10_ Reduction (Mean ± SD)	Number of Culture-Positive Results per Total Number of Experiments	*p* vs. 0 MPa
Planktonic *S. aureus* (35556)	0.9% saline	0 (control)	0	7/7	-
		250	−0.007 ± 0.140	7/7	0.9997
		350	0.377 ± 0.205	7/7	0.5378
	0.0031% SDS/0.31 mM EDTA	0 (control)	0.163 ± 0.091	7/7	-
		250	1.407 ± 0.982	7/7	*0.0046*
		350	1.579 ± 1.032	7/7	*0.0012*
	0.0063% SDS/0.63 mM EDTA	0 (control)	1.987 ± 0.985	6/6	-
		250	5.043 ± 1.338	6/6	*<0.0001*
		350	5.620 ± 1.671	6/6	*<0.0001*
	0.0125% SDS/1.25 mM EDTA	0 (control)	6.487 ± 0.690	1/7	-
		250	6.656 ± 0.191	1/7	0.8777
		350	6.399 ± 0.812	1/7	0.9642
	0.05% SDS/5 mM EDTA	0 (control)	**6.741 ± 0.187**	0/7	-
		250	**6.741 ± 0.187**	0/7	>0.999
		350	**6.741 ± 0.187**	0/7	>0.999
Planktonic *S. epidermidis* (35984)	0.9% saline	0 (control)	0	4/4	-
		250	4.730 ± 0.600	2/3	*<0.0001*
		350	**5.443 ± 0.390**	0/3	*<0.0001*
	0.0031% SDS/0.31 mM EDTA	0 (control)	0.190 ± 0.253	4/4	-
		250	4.858 ±1.214	1/4	*<0.0001*
		350	**5.443 ± 0.390**	0/3	*<0.0001*
	0.0063% SDS/0.63 mM EDTA	0 (control)	1.440 ± 0.290	3/3	-
		250	5.217 ± 0.625	1/3	*<0.0001*
		350	**5.553 ± 0.384**	0/3	*<0.0001*
	0.0125% SDS/1.25 mM EDTA	0 (control)	3.770 ± 1.033	3/4	-
		250	**5.553 ± 0.384**	0/3	*<0.0001*
		350	**5.553 ± 0.384**	0/3	*<0.0001*
	0.05% SDS/ 5 mM EDTA	0 (control)	**5.553 ± 0.384**	0/3	-
		250	**5.553 ± 0.384**	0/3	>0.9999
		350	**5.553 ± 0.384**	0/3	>0.9999
Planktonic *S. epidermidis* (12228)	0.9% saline	0 (control)	0	3/3	-
		250	3.623 ± 0.355	3/3	*<0.0001*
		350	**6.660 ± 0.270**	0/3	*<0.0001*
	0.0031% SDS/ 0.31 mM EDTA	0 (control)	0.277 ± 0.430	3/3	-
		250	5.840 ± 0.589	2/3	*<0.0001*
		350	**6.660 ± 0.270**	0/3	*<0.0001*
	0.0063% SDS/ 0.63 mM EDTA	0 (control)	1.643 ± 1.082	3/3	-
		250	**6.660 ± 0.270**	0/3	*<0.0001*
		350	**6.660 ± 0.270**	0/3	*<0.0001*
	0.0125% SDS/ 1.25 mM EDTA	0 (control)	5.287 ± 1.586	3/3	-
		250	**6.660 ± 0.270**	0/3	*0.0126*
		350	**6.660 ± 0.270**	0/3	*0.0126*
	0.05% SDS/ 5 mM EDTA	0 (control)	**6.660 ± 0.270**	0/3	-
		250	**6.660 ± 0.270**	0/3	>0.9999
		350	**6.660 ± 0.270**	0/3	>0.9999

EDTA, ethylenediaminetetraacetic acid; SD, standard deviation; SDS, sodium dodecyl sulfate.

**Table 4:** Detailed log_10_ reductions and statistical comparisons for biofilm-associated *S. aureus* and *S. epidermidis* under varying SDS/EDTA concentrations and pressure levels. Bold log_10_ reduction values: maximum achievable log reduction, logRF_max_; italic *p*-values: statistically significant vs. non-pressurized controls.

Sample Organism (ATCC)	Pressure-Transmitting Medium	Pressure Level [MPa]	log_10_ Reduction (Mean ± SD)	Number of Culture-Positive Results per Total Number of Experiments	*p* vs. 0 MPa
Biofilm-associated *S. aureus* (35556)	0.9% saline	0 (control)	0	4/4	-
		250	0.030 ± 0.129	4/4	0.9985
		350	0.655 ± 0.189	4/4	0.5010
	0.0125% SDS/1.25 mM EDTA	0 (control)	0.543 ± 0.332	4/4	-
		250	0.910 ± 0.509	4/4	0.7990
		350	2.050 ± 0.589	3/3	0.0667
	0.05% SDS/5 mM EDTA	0 (control)	5.374 ± 1.373	2/5	-
		250	6.110 ± 1.770	1/4	0.9203
		350	**6.798 ± 0.469**	0/4	0.3471
Biofilm-associated *S. epidermidis* (35984)	0.9% saline	0 (control)	0	3/3	-
		250	1.150 ± 0.610	3/3	0.0761
		350	2.255 ± 1.803	2/2	*0.0014*
	0.0125% SDS/1.25 mM EDTA	0 (control)	0.850 ± 0.496	3/3	-
		250	1.897 ± 0.771	3/3	0.1106
		350	5.000 ± 0.494	3/3	*<0.0001*
	0.05% SDS/5 mM EDTA	0 (control)	5.507 ± 1.464	1/3	-
		250	**6.477 ± 0.294**	0/3	0.1455
		350	**6.477 ± 0.294**	0/3	0.1455

EDTA, ethylenediaminetetraacetic acid; SD, standard deviation; SDS, sodium dodecyl sulfate.

## Data Availability

The original contributions presented in this study are included in the article. Further inquiries can be directed to the corresponding author.

## Abbreviations

The following abbreviations are used in this manuscript:

ATCC	American Type Culture Collection
CFU	colony forming units
CRC	checkerboard reference concentration
EPS	extracellular polymeric substances
EDTA	ethylenediaminetetraacetic acid
FIC	fractional inhibitory concentration
HHP	high hydrostatic pressure
logRF	logarithmic reduction factor
logRF_max_	maximum achievable log reduction
MIC	minimum inhibitory concentration
MIC_comb_	minimum inhibitory concentration of the additive combination
MPa	Megapascal
PBS	phosphate-buffered saline
SD	standard deviation
SDS	sodium dodecyl sulfate
SEM	scanning electron microscopy
TSB	tryptic soy broth

## Appendix A

**Table A1:** Viable bacteria counts of *S. aureus* ATCC 35556 under varying SDS/EDTA concentrations over 3 h. CFU values are displayed as CFU/mL × 10^3^. Bold CFU values: maximum reduction.

Pressure-Transmitting Medium	Time [h]	Viable Count ± SD [CFU/mL] × 10^3^	Min to Max Range [CFU/mL] × 10^3^	Number of Culture-Positive Results per Total Number of Experiments
0.9% saline control	0	154,940 ± 63,313	87,100–224,000	5/5
0.9% saline	0.25	149,080 ± 59,951	84,000–210,000	5/5
	1	111,880 ± 37,286	56,000–152,000	5/5
	2	167,960 ± 80,493	79,000–252,000	5/5
	3	127,640 ± 54,510	44,800–172,000	5/5
0.0031% SDS/0.31 mM EDTA	0.25	118,560 ± 33,848	74,800–167,000	5/5
	1	101,200 ± 28,560	56,000–134,000	5/5
	2	120,120 ± 43,663	64,400–154,000	5/5
	3	140,980 ± 81,186	86,800–274,000	5/5
0.0063% SDS/0.63 mM EDTA	0.25	44,360 ± 32,674	17,100–98,500	5/5
	1	53,560 ± 46,991	14,600–135,000	5/5
	2	43,376 ± 56,105	3040–141,000	5/5
	3	39,030 ± 39,687	9520–106,000	5/5
0.0125% SDS/1.25 mM EDTA	0.25	3.07 ± 1.92	0–5.10	4/5
	1	2.52 ± 1.70	0–4.41	4/5
	2	14.8 ± 22.7	0.31–54.6	5/5
	3	2.72 ± 2.53	0–6.36	4/5
0.05% SDS/5 mM EDTA	0.25	**0.00 ± 0.00**	–	0/5
	1	**0.00 ± 0.00**	–	0/5
	2	**0.00 ± 0.00**	–	0/5
	3	**0.00 ± 0.00**	–	0/5

CFU, colony forming units; EDTA, ethylenediaminetetraacetic acid; SD, standard deviation; SDS, sodium dodecyl sulfate.

**Table A2:** Viable bacteria counts of planktonic *S. aureus* and *S. epidermidis* under varying SDS/EDTA concentrations and pressure levels. CFU values are displayed as CFU/mL × 10^3^. Bold CFU values: maximum reduction.

Sample Organism (ATCC)	Pressure-Transmitting Medium	Pressure Level [MPa]	Viable Count ± SD [CFU/mL] × 10^3^	Min to Max Range [CFU/mL] × 10^3^	Number of Culture-Positive Results per Total Number of Experiments
Planktonic *S. aureus* (35556)	0.9% saline	0 (control)	334,143 ± 139,933	174,000–552,000	7/7
		250	351,571 ± 163,531	137,000–568,000	7/7
		350	156,400 ± 109,667	72,200–372,000	7/7
	0.0031% SDS/ 0.31 mM EDTA	0 (control)	230,000 ± 107,793	134,000–440,000	7/7
		250	79,577 ± 11,028	560–282,000	7/7
		350	41,475 ± 59,474	84–158,000	7/7
	0.0063% SDS/ 0.63 mM EDTA	0 (control)	10,008 ± 8994	28–23,500	6/6
		250	13.2 ± 13.0	0–27.3	6/6
		350	34.7 ± 58.3	0–140	6/6
	0.0125% SDS/ 1.25 mM EDTA	0 (control)	0.48 ± 1.27	0–3.36	1/7
		250	0.03 ± 0.08	0–0.22	1/7
		350	2.01 ± 5.33	0–14.1	1/7
	0.05% SDS/ 5 mM EDTA	0 (control)	**0.00 ± 0.00**	–	0/7
		250	**0.00 ± 0.00**	–	0/7
		350	**0.00 ± 0.00**	–	0/7
Planktonic *S. epidermidis* (35984)	0.9% saline	0 (control)	32,990 ± 29,199	7560–70,000	4/4
		250	1.34 ± 2.23	0–3.92	2/3
		350	**0.00 ± 0.00**	–	0/3
	0.0031% SDS/ 0.31 mM EDTA	0 (control)	30,310 ± 41,841	4480–92,400	4/4
		250	14.0 ± 28.0	0–56	1/4
		350	**0.00 ± 0.00**	–	0/3
	0.0063% SDS/ 0.63 mM EDTA	0 (control)	2655 ± 3693	273–6910	3/3
		250	0.53 ± 0.92	0–1.60	1/3
		350	**0.00 ± 0.00**	–	0/3
	0.0125% SDS/ 1.25 mM EDTA	0 (control)	38.6 ± 62.4	1.34–131	3/4
		250	**0.00 ± 0.00**	–	0/3
		350	**0.00 ± 0.00**	–	0/3
	0.05% SDS/ 5 mM EDTA	0 (control)	**0.00 ± 0.00**	–	0/3
		250	**0.00 ± 0.00**	–	0/3
		350	**0.00 ± 0.00**	–	0/3
Planktonic *S. epidermidis* (12228)	0.9% saline	0 (control)	291,667 ± 197,130	168,000–519,000	3/3
		250	115 ± 147	17.9–284	3/3
		350	**0.00 ± 0.00**	–	0/3
	0.0031% SDS/ 0.31 mM EDTA	0 (control)	142,833 ± 64,634	93,500–216,000	3/3
		250	0.63 ± 0.54	0–0.98	2/3
		350	**0.00 ± 0.00**	–	0/3
	0.0063% SDS/ 0.63 mM EDTA	0 (control)	24,253 ± 39,098	1620–69,400	3/3
		250	**0.00 ± 0.00**	–	0/3
		350	**0.00 ± 0.00**	–	0/3
	0.0125% SDS/ 1.25 mM EDTA	0 (control)	20.6 ± 35.3	0.17–61.3	3/3
		250	**0.00 ± 0.00**	–	0/3
		350	**0.00 ± 0.00**	–	0/3
	0.05% SDS/ 5 mM EDTA	0 (control)	**0.00 ± 0.00**	–	0/3
		250	**0.00 ± 0.00**	–	0/3
		350	**0.00 ± 0.00**	–	0/3

CFU, colony forming units; EDTA, ethylenediaminetetraacetic acid; SD, standard deviation; SDS, sodium dodecyl sulfate.

**Table A3:** Viable bacteria counts of biofilm-associated *S. aureus* and *S. epidermidis* under varying SDS/EDTA concentrations and pressure levels. CFU values are displayed as CFU/mL × 10^3^. Bold CFU values: maximum reduction.

Sample Organism (ATCC)	Pressure-Transmitting Medium	Pressure Level [MPa]	Viable Count ± SD [CFU/mL] × 10^3^	Min to Max Range [CFU/mL] × 10^3^	Number of Culture-Positive Results per Total Number of Experiments
Biofilm-associated *S. aureus* (35556)	0.9% saline	0 (control)	615,500 ± 416,999	241,000–1,210,000	4/4
		250	557,250 ± 297,474	199,000–899,000	4/4
		350	124,100 ± 53,494	68,000–185,000	4/4
	0.0125% SDS/ 1.25 mM EDTA	0 (control)	150,500 ± 25,173	126,000–185,000	4/4
		250	81,500 ± 61,997	23,500–165,000	4/4
		350	7277 ± 4413	2270–10,600	3/3
	0.05% SDS/ 5 mM EDTA	0 (control)	104 ± 121	0–280	2/5
		250	7.95 ± 15.9	0–31.8	1/4
		350	**0.00 ± 0.00**	–	0/4
Biofilm-associated *S. epidermidis* (35984)	0.9% saline	0 (control)	193,967 ± 114,063	80,900–309,000	3/3
		250	25,773 ± 34,137	2800–65,000	3/3
		350	16,228 ± 22,871	56–32,400	2/2
	0.0125% SDS/ 1.25 mM EDTA	0 (control)	35,860 ± 35,717	7280–75,900	3/3
		250	3074 ± 2898	2230–6920	3/3
		350	2.05 ± 1.48	0.84–3.7	3/3
	0.05% SDS/ 5 mM EDTA	0 (control)	15.4 ± 26.6	0–46.1	1/3
		250	**0.00 ± 0.00**	–	0/3
		350	**0.00 ± 0.00**	–	0/3

CFU, colony forming units; EDTA, ethylenediaminetetraacetic acid; SD, standard deviation; SDS, sodium dodecyl sulfate.
